# Changing trends of suicide mortality from 2011 to 2019: an analysis of 38 European Countries

**DOI:** 10.1192/j.eurpsy.2023.264

**Published:** 2023-07-19

**Authors:** G. Fico, A. Gimenez-Palomo, R. Andra Bursan, C. R. Ionescu, F. Kraxner, P. Rolland, S. Gomes-Rodrigues, M. Batković, E. Metaj, S. Tanyeri Kayahan, A. Mamikonyan, P. Paribello, A. K. Sikora, C. M. Platsa, M. Spasic Stojakovic, A. H. Halt, M. Az, N. Ovelian, K. Melamud, M. Janusz, K. Hinkov, C. Gramaglia, J. Beezhold, J. L. Castroman, C. Hanon, D. Eraslan, E. Olie

**Affiliations:** 1 Bipolar and Depressive Disorders Unit - Hospital Clinic of Barcelona - IDIBAPS, Barcelona, Spain; 2 “Alexandru Obregia” Clinical Psychiatry Hospital, Bucarest, Romania; 3 Department of psychiatry and psychotherapy, Clinical Hospital Affoltern, Zurich, Switzerland; 4 Academic Psychiatry Department, Centre Hospitalier Guillaume Regnier, Rennes, France; 5 Child and Adolescent Psychiatry Department, Centro Hospitalar e Universitário do Porto, Porto, Portugal; 6 Psychiatry Department, General Hospital, Dubrovnik, Croatia; 7 Mia Clinic, Tirana, Albania; 8 Psychiatry, Turkish Ministry of Health Yalvac Public Hospital, Isparta, Türkiye; 9 Psychiatry, Yerevan State Medical University after Mkhitar Heratsi, Yerevan, Armenia; 10 Department of Medical Sciences and Public Health, University of Cagliari, Cagliari, Italy; 11 Addictions and Medical Psychology, Ivano-Frankivsk National Medical University, Ivano-Frankivsk, Ukraine; 12 Psychiatric Hospital of Thessaloniki, Thessaloniki, Greece; 13 Institute of Mental Health, Belgrade, Serbia; 14 University of Oulu, Research Unit of Clinical Medicine; 15 Department of Psychiatry, Oulu University Hospital, Oulu, Finland; 16 State Hospital of Abdulkadir Yuksel, Gaziantep, Türkiye; 17 Saint Petersburg State Mental Hospital, Saint Petersburg, Russian Federation; 18 Kharkiv Psychoneurological dispensary, Kharkiv, Ukraine; 19 Instytut Psychiatrii i Neurologii w Warszawie, Warsaw, Poland; 20 Etableesement Public de santé Alsace Nord, Strasbourg, France; 21 Università del Piemonte Orientale, Maggiore della Carità University Hospital, Novara, Italy; 22 Great Yarmouth Acute Service, Northgate Hospital, Great Yarmouth, United Kingdom; 23 Department of Psychiatry, Nimes University Hospital, France, IGF, Univ. Montpellier, CNRS, INSERM, Montpellier; 24 Centre ressource régional de psychiatrie du sujet âgé (CRRPSA), AP-HP, Centre-Université de Paris, Paris, France; 25 Istanbul Psikiyatri Enstitusu, Istanbul, Türkiye; 26 Department of Emergency Psychiatry and Acute Care, Lapeyronie Hospital, IGF, Univ. Montpellier, CNRS, INSERM, Montpellier, France

## Abstract

**Introduction:**

Suicide is a serious public health problem since it accounts for nearly 900,000 deaths each year worldwide. Globally in 2019, 10.7 persons out of 100,000 died by suicide. Psychiatric disorders are related to an overwhelming proportion of these cases. In the last years, several specific interventions and action plans for suicide prevention have been implemented in a number of European countries.

**Objectives:**

Our aim was to analyze recent epidemiologic trends of suicide mortality rates in Europe.

**Methods:**

Annual national statistics of suicide mortality rates derived from Eurostat public databases from 2011 to 2019 were analyzed for 38 European countries. The suicide mortality rate was estimated per year/100,000 population. Linear regression models were used to study temporal trends of suicidal mortality. Analyses were performed using RStudio.

**Results:**

Available data show a statistically significant reduction in suicide mortality rates from 2011 to 2019 in 15 European countries, and a significant increase for Turkey (ES=0.32, SD=0.06, p=0.037) (Fig 1). The greatest significant decrease was reported in Lithuania (ES=-1.42, SD=0.02, p=0.02), followed by Hungary (ES=-1.13, SD=0.11, p=0.0007), Latvia (ES=-0.76, SD=0.11, p=0.007), and Poland (ES=-0.73, SD=0.10, p=0.001). Italy reported the lowest significant reduction in suicide mortality rates (ES=-0.13, SD=0.018, p=0.003). The remaining 16 countries showed no significant changes in suicide mortality trends.

**Image:**

**Figure:**
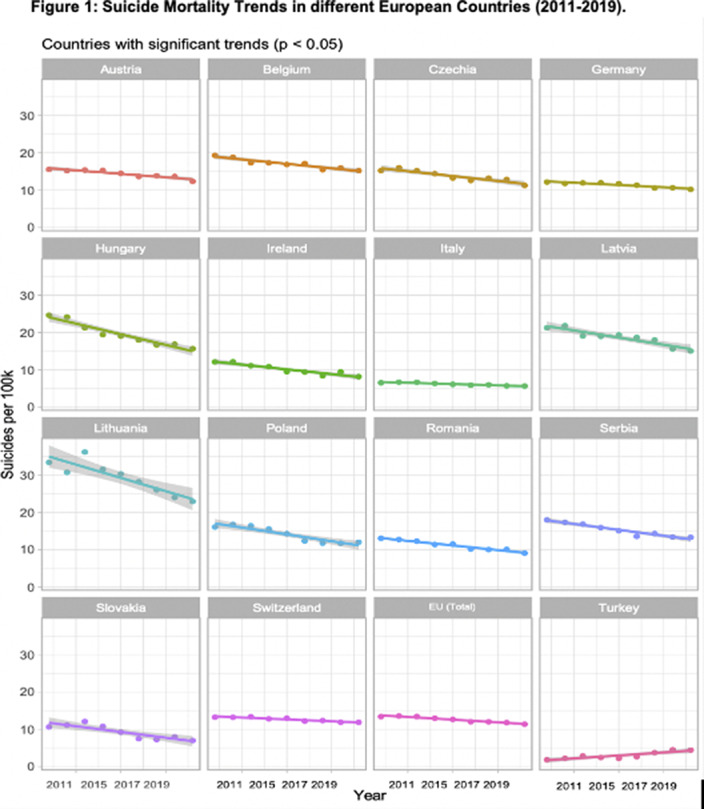

**Conclusions:**

In the last years, Europe registered an overall reduction in reported suicide rates. However, more recent data (i.e., suicide rates after COVID-19 pandemic, age and sex-related effect on suicide rates) should be analyzed and used to implement future recommendations. Current and future suicide prevention strategies aim to contribute to a greater reduction of suicide rates in the different European countries.

**Disclosure of Interest:**

None Declared

